# Reframing Older Adults’ Violence Toward Staff as Self-Protection

**DOI:** 10.1093/geroni/igab046.1270

**Published:** 2021-12-17

**Authors:** Raelene Shippee-Rice, Rosemary Taylor, Lisa Mistler, Pamela DiNapoli, Karla Armenti

**Affiliations:** 1 University of New Hampshire, Nottingham, New Hampshire, United States; 2 University of Massachusetts Medical School, Worcester, Massachusetts, United States; 3 Geisel School of Medicine; Dartmouth-Hitchcock Health; New Hampshire Hospital, Concord, New Hampshire, United States; 4 University of New Hampshire, Durham, New Hampshire, United States

## Abstract

One of the first studies on workplace violence in nursing homes was published in 1985. Forty-five (45) years later, resident violence against staff continues to increase in incidence and severity. At the request of a state senator, a New Hampshire psychiatrist formed a research group to conduct the first New Hampshire survey on staff experience of workplace violence. Study questions focused on experiences of workplace violence and incident reporting, and the availability and benefit of workplace violence training programs. Results were consistent with recently published literature: violence is an expected, normalized element when providing care; potential repercussions and perceived resident lack of intent were major reasons for incident non-reporting. Analysis of study results and review of the literature led to the question: Are older residents’ violent behaviors towards staff an act of self-protection?

